# Effectiveness of clinical dashboards as audit and feedback or clinical decision support tools on medication use and test ordering: a systematic review of randomized controlled trials

**DOI:** 10.1093/jamia/ocac094

**Published:** 2022-06-11

**Authors:** Charis Xuan Xie, Qiuzhe Chen, Cesar A Hincapié, Léonie Hofstetter, Chris G Maher, Gustavo C Machado

**Affiliations:** Wolfson Institute of Population Health, Barts and The London School of Medicine and Dentistry, Queen Mary University of London, London, UK; Institute for Musculoskeletal Health, Sydney, NSW, Australia; Sydney School of Public Health, Faculty of Medicine and Health, The University of Sydney, Sydney, NSW, Australia; Department of Chiropractic Medicine, Faculty of Medicine, University of Zurich and Balgrist University Hospital, Zurich, Switzerland; Epidemiology, Biostatistics and Prevention Institute, University of Zurich, Zurich, Switzerland; Department of Chiropractic Medicine, Faculty of Medicine, University of Zurich and Balgrist University Hospital, Zurich, Switzerland; Institute for Musculoskeletal Health, Sydney, NSW, Australia; Sydney School of Public Health, Faculty of Medicine and Health, The University of Sydney, Sydney, NSW, Australia; Institute for Musculoskeletal Health, Sydney, NSW, Australia; Sydney School of Public Health, Faculty of Medicine and Health, The University of Sydney, Sydney, NSW, Australia

**Keywords:** dashboard, audit and feedback, clinical decision support, review

## Abstract

**Background:**

Clinical dashboards used as audit and feedback (A&F) or clinical decision support systems (CDSS) are increasingly adopted in healthcare. However, their effectiveness in changing the behavior of clinicians or patients is still unclear. This systematic review aims to investigate the effectiveness of clinical dashboards used as CDSS or A&F tools (as a standalone intervention or part of a multifaceted intervention) in primary care or hospital settings on medication prescription/adherence and test ordering.

**Methods:**

Seven major databases were searched for relevant studies, from inception to August 2021. Two authors independently extracted data, assessed the risk of bias using the Cochrane RoB II scale, and evaluated the certainty of evidence using GRADE. Data on trial characteristics and intervention effect sizes were extracted. A narrative synthesis was performed to summarize the findings of the included trials.

**Results:**

Eleven randomized trials were included. Eight trials evaluated clinical dashboards as standalone interventions and provided conflicting evidence on changes in antibiotic prescribing and no effects on statin prescribing compared to usual care. Dashboards increased medication adherence in patients with inflammatory arthritis but not in kidney transplant recipients. Three trials investigated dashboards as part of multicomponent interventions revealing decreased use of opioids for low back pain, increased proportion of patients receiving cardiovascular risk screening, and reduced antibiotic prescribing for upper respiratory tract infections.

**Conclusion:**

There is limited evidence that dashboards integrated into electronic medical record systems and used as feedback or decision support tools may be associated with improvements in medication use and test ordering.

## INTRODUCTION

With the widespread uptake of electronic medical records, massive health-related datasets have been generated and they continue to grow at unprecedented rates.^1^^,^^2^ Despite the potential impact of using these datasets to improve patient care, clinicians are often overwhelmed with the complexity of processing electronic medical record data.^3^^,^^4^ To better utilize these routinely collected data, clinical dashboards have been developed and integrated into electronic medical record systems to help clinicians make informed decisions and ensure the quality and safety of the care delivered.^1^^,^^2^

Clinical dashboards are interactive data visualization tools that provide a visual summary of decision-related clinical information displayed in graphs, charts, or interactive tables.^5^ They are commonly used in healthcare as clinical decision support systems (CDSS) or audit and feedback (A&F) tools to help clinicians make informed decisions and to provide feedback on variations in care. Clinical dashboards integrated into electronic medical record systems can display critical indicators to clinicians allowing them to recognize suboptimal care, which has been used to motivate better performance.^6^^,^^7^ Suboptimal care related to medication prescription and test ordering has been extensively reported in the literature. Examples of this include overuse of medications (such as antibiotics and opioid analgesics)^8^ and unnecessary referrals for diagnostic imaging or laboratory tests,^9^ despite numerous guidelines endorsing rational use of these interventions. Optimizing medication prescription and test ordering are central to high-quality healthcare and clinical dashboards are therefore promising tools for enabling clinicians to reflect on their practice and identify areas to change.

Traditional methods of CDSS and A&F without the use of clinical dashboards have been shown to improve healthcare delivery. For instance, a recent systematic review revealed that CDSS integrated into electronic medical record systems increased the proportion of patients receiving desired care by 5.8% compared with usual care.^10^ A Cochrane review showed that A&F interventions resulted in a 4.3% absolute increase in healthcare professionals’ compliance with the desired practice.^11^ None of these reviews, however, considered clinical dashboards as CDSS and A&F mechanisms, despite the increasing use in the last decade. A previous narrative review of 11 studies on the effects of clinical dashboards included only 1 randomized controlled trial, which showed no effect on antibiotic prescribing for acute respiratory infection in primary care.^5^ This review is now 9 years old and did not conduct systematic searches nor assess the risk of bias. A more recent systematic review^12^ focused on critical care units and included a wide range of data visualization techniques, that is, not only clinical dashboards.

Despite the increasing popularity in healthcare, there is limited knowledge on the effectiveness of clinical dashboards in changing clinician or patient’s behavior. In this systematic review, we aimed to assess the effectiveness of clinical dashboards used as CDSS or A&F tools (as a standalone intervention or part of a multifaceted intervention) in primary care or hospital settings on medication prescription, adherence, and test ordering.

## METHODS

This systematic review followed the Preferred Reporting Items for Systematic Reviews and Meta-Analyses (PRISMA) recommendations.^13^ The protocol has been published in the Open Science Framework.^14^

### Searches

Electronic literature searches were conducted in the following databases: MEDLINE via Ovid, EMBASE via Ovid, and CINAHL via EBSCO, CENTRAL via Cochrane Library, INSPEC, ACM Digital Library, and IEEEXplore, from inception to August 2021. We combined the following terms and their variations to construct the search strategies: dashboard, decision support, electronic health record, and quality indicators. The reference lists of included studies and relevant systematic reviews were screened for additional relevant citations. We did not restrict our searches to any language or date of publication. The search strategies used for the selected databases are outlined in Supplementary File S1.

Titles and abstracts of records retrieved from our electronic searches were screened independently by 2 reviewers. Full texts of potentially eligible articles were screened independently by 2 reviewers according to the eligibility criteria, and disagreements were resolved by consensus or in consultation with a third reviewer.

### Eligibility criteria

#### Types of studies

Eligible studies had to be randomized controlled trials published in peer-reviewed journals. In our protocol, we stated we would consider observational studies (eg, prospective or retrospective cohorts) but we later decided to include only randomized controlled trials, which allowed us to focus on the highest level of evidence to investigate the effects of dashboard interventions. Conference abstracts and study protocols were excluded.

#### Types of participants and settings

Studies including clinicians or patients as participants, investigating any health condition in primary care or hospital settings (eg, emergency departments, hospital wards, and outpatient clinics) were considered. Studies including healthy populations or healthcare students were excluded.

#### Types of interventions and comparators

We considered clinical dashboard interventions used as CDSS or A&F. Clinical dashboards included those involving graphical user interfaces containing measures of clinical performance or clinical indicators to enable decision-making. We also considered clinical dashboards that provided a visual summary of decision-related information displayed in graphs, charts, or interactive tables. We also included studies with multifaceted/multicomponent interventions including a clinical dashboard as a core component. Studies comparing the effectiveness of clinical dashboard interventions with any type of control were considered, including usual care, no intervention, and a similar intervention without the dashboard component.

#### Types of outcome measures

The 2 outcomes of interest for this review were: (1) medication use, including the rate of medication prescribed/administered and medication intake adherence; (2) test ordering, such as the rate of imaging referrals and the count of routine laboratory test orders. We focused on these outcomes (rather than clinical or patient-reported outcomes) as we aimed to evaluate whether clinical dashboards with CDSS or A&F features achieve their main objective—changing clinician or patient’s behavior.

### Data extraction

A standardized spreadsheet was developed, and 2 reviewers independently extracted the data from the included studies. The extracted data from the included studies were: study design, sample size, sample characteristics (source, health condition, age, sex), healthcare setting, country, type of dashboard, dashboard features, intervention characteristics, outcome measures, and time points. The effect sizes (eg, mean difference, risk ratio, and odds ratio) and their 95% confidence intervals (CIs) were also extracted.

### Risk of bias assessment

The Cochrane Risk of Bias (RoB) 2.0 tool^15^ was used to assess the risk of bias of included randomized controlled studies. The risk of bias of each domain was judged as high risk of bias, low risk of bias, and some concerns, and overall risk of bias for each included study was also provided. Studies were considered as having an overall low risk of bias when all domains were judged as low risk, whereas studies were considered as having an overall high risk of bias when at least 1 bias domain was judged as high risk.^15^ For studies with some concerns in at least 1 domain, we recorded it as having some concerns.

### Certainty of evidence

The Grading of Recommendations of Assessment, Development and Evaluation (GRADE) approach^16^ was used to assess the quality of the body of evidence for the primary outcomes of included studies. The GRADE ratings were summarized as either high, moderate, low, or very low across 5 domains: study limitations, inconsistency of results, indirectness of evidence, imprecision of estimates and publication bias.^16^ Since we did not conduct a meta-analysis, assessment against inconsistency and publication bias was not applicable in this review.

### Data synthesis

Since high heterogeneity exists in health conditions and outcome measures, we were unable to perform a meta-analysis. Therefore, a narrative synthesis of the findings was conducted. We descriptively reported the effect sizes for both primary and secondary outcomes related to medication use and test ordering, the intervention time frame, and the number of participants in each trial. Relevant data were grouped and assessed based on the types of interventions (ie, CDSS, A&F, standalone, or multifaceted interventions) and types of comparators (eg, usual care/no intervention). Results are presented in the summary of findings tables along with the GRADE assessment.

## RESULTS

**Figure 1.**
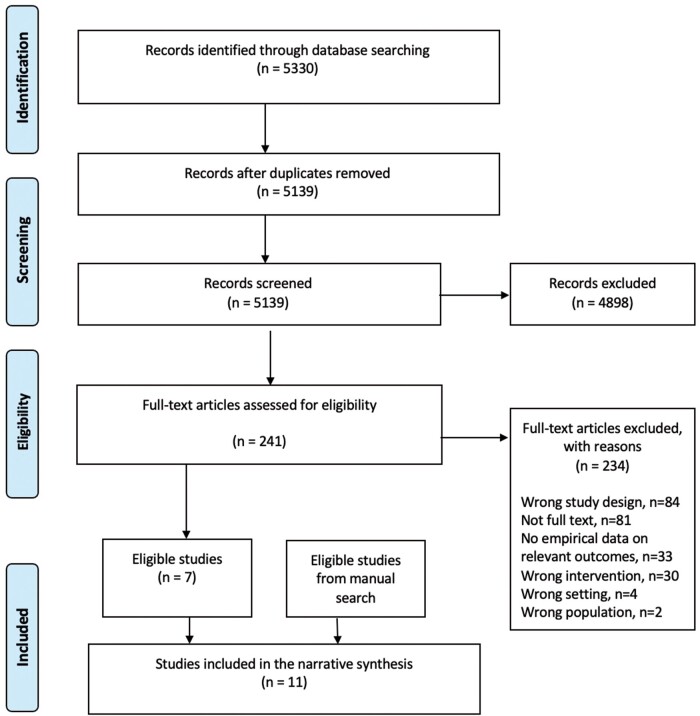
PRISMA flow diagram. The PRIMSA flow diagram presents the systematic search and selection process in this review, detailing the number of records included and excluded at different stages and showing the final number of included studies.

In total, after the removal of duplicates, there were 5139 studies screened by title and abstract, of which 4898 were deemed not to be relevant, leaving 241 studies for full-text screening. Of those full-text studies, 7 studies were eligible randomized controlled trials. Four additional randomized controlled trials were found via reference screened of included studies. We included 11 trials in this systematic review. The PRISMA flowchart is shown in Figure 1.

### Study characteristics

Table 1 provides a full description of the study characteristics (including key dashboard types and features). Of the 11 randomized controlled trials, 7 were cluster randomized trials,^17^^,^^18^^,^^20–22^^,^^25^^,^^27^ while 4 trials were 2-arm parallel randomized trials.^19^^,^^23^^,^^24^^,^^26^ Four trials were conducted in the United States,^17^^,^^18^^,^^20^^,^^23^ while another 4 studies were from other Asia-Pacific countries (Australia, China, and South Korea),^21^^,^^22^^,^^24^^,^^25^ and 3 studies were from European countries (United Kingdom, Switzerland).^19^^,^^26^^,^^27^ Six studies were conducted in primary care^17^^,^^18^^,^^23^^,^^25–27^ while 5 studies were done in hospital settings.^19–22^^,^^24^ The dashboard interventions targeted mainly clinician participants (n = 9) and 2 studies used the dashboard interventions in patients.^19^^,^^24^

**Table 1:** Characteristics of the included studies

Study	Design	Setting	Dashboard types	Dashboard features	Outcomes
Linder et al^17^ USA	Cluster randomized controlled trial	27 primary care practices	*Feedback dashboard* Quality dashboard displaying antibiotic prescribing and billing practices data for acute respiratory infections	Bar graph displayed clinician’s prescribing rates for acute respiratory infections for previous year (data updated monthly) vs clinic peers and national benchmarks. Dashboard allowed clinicians to “drill down” to view any individual patient medical record.	Primary: antibiotic prescribing rate for all acute respiratory infection visits. Secondary: antibiotic prescribing rate for: (1) antibiotic-appropriate acute respiratory infection visits and (2) non–antibiotic-appropriate acute respiratory infection visits.
Patel et al^18^ USA	Cluster randomized controlled trial	32 primary care clinics at the University of Pennsylvania Health System	*Feedback dashboard* An automated patient dashboard listing patients who met national guidelines for statin therapy but had not been prescribed this medication.	Dashboard linked to the American College of Cardiology/American Heart Association guidelines, showed options for selecting statin dosage. Dashboard provided clinicians a list of patients who met guidelines for statin therapy but have not been prescribed, to be reviewed in 1 wk. Also provided clinician performance feedback based on baseline statin prescribing rates and compared with peers. Data were obtained from patient electronic health records.	Primary: statin prescribing rates for atherosclerotic cardiovascular disease in dashboard only group and dashboard with peer comparison group.
EI Miedany et al^19^ Europe	Randomized controlled trial	Not reported	*Feedback dashboard* A visual feedback tool in the management of rheumatology	Dashboard enabled patients to monitor real-time changes of their disease activity parameters and patient’s reported outcome measures. Electronic data recording in the standard rheumatology clinical practices were integrated in the visual feedback system.	Primary: the change in the patients’ adherence to medications.
Ryskina et al^20^ USA	Cluster randomized controlled trial	Hospital of the University of Pennsylvania	*Feedback dashboard* A personalized, EMR-based, real-time dashboard containing patient level details for internal medicine residents	Dashboard provided the internal medicine residents with feedback on their use of routine laboratory tests relative to service averages. Dashboard contained real-time lab ordering information which was linked to individual patients’ EMR records.	Primary outcome: the count of routine laboratory test orders placed by a physician per patient-day.
Coombs et al^21^ Australia	Stepped-wedge, cluster-randomized trial	4 EDs in New South Wales, Australia	*Multifaceted intervention incorporating a dashboard component* Real-time dashboard developed in Qlik Sense for clinicians	Dashboard provided clinicians with structured real-time audit and feedback data on department-level imaging, opioid and inpatient admission rates. Dashboard was integrated into the electronic medical record system.	Primary outcome: the proportion of low back pain presentations receiving lumbar imaging. Secondary outcomes: healthcare utilization outcomes included prescriptions of pain medicines.
Chang et al^22^ China	Cluster randomized crossover open controlled trial	31 township public hospitals	*Feedback dashboard* A computer network-based feedback dashboard for physicians	Dashboard displayed physicians’ antibiotic prescription rates, frequency and ranking updated every 10 d. Dashboard presented top 5 diseases of patients, number of prescriptions, antibiotic frequency and prescription rate, precautions and contraindications for antibiotics being use. Enabled pop-up window to automatically prompt physicians to check for the feedback information every 10 d.	Primary outcome: 10-d antibiotic prescription rate of physicians (defined as the number of antibiotic prescriptions divided by the total number of the prescriptions in each 10-d time period).
Du et al^23^ USA	Randomized controlled trial	A telemedicine practice	*Multifaceted intervention incorporating a dashboard component* Individualized prescribing feedback dashboards for clinicians	Dashboard displayed monthly rates of personal and practice-wide antibiotic prescription rates starting May 2018 and summarized antibiotic prescription rates for the previous month. Data were collected from patient electronic health records.	Primary outcome: antibiotic prescription rates for each of the 4 diagnostic categories: upper respiratory infection, bronchitis, sinusitis, and pharyngitis.
Jung et al^24^ South Korea	Randomized controlled trial	A university hospital	*Feedback dashboard* ICT-based centralized monitoring dashboard for increasing medication adherence among kidney transplant recipients	The ICT based centralized monitoring system alerted both patients and medical staff with texts and pill box alarms.	Primary outcome: medical adherence among kidney transplant recipients.
Peiris et al^25^ Australia	Cluster Randomized Trial	60 primary healthcare centers	*Multifaceted intervention incorporating a dashboard component* Cardiovascular disease risk management dashboard	Dashboard allowed health services to audit health records, identify performance gaps, and establish recall/reminder prompts rapidly. Dashboard used traffic light prompts to alert the practitioner to suggest recommendations.	Primary outcomes: (1) the proportion of eligible patients who received appropriate screening of CVD risk factors and (2) the proportion of eligible patients defined at baseline as being at high CVD risk receiving recommended medication prescriptions at the end of study. Secondary outcomes (1) escalation of drug prescription among patients at high CVD risk (either newly prescribed or additional numbers of antiplatelet, BP-lowering, and lipid-lowering agents).
Hemkens et al^26^ Switzerland	Randomized controlled trial	National-wide primary care practices	*Feedback dashboard* Personalized prescription feedback dashboard for physicians	Dashboard displayed of quarterly updated single-page graphical overview (bar chart) showing individual amount of antibiotic prescriptions per 100 consultations in the preceding months and the adjusted average in peer physicians across national-wide physician population.	Primary outcome: the prescribed defined daily doses (DDD) of any type of antibiotics to any patient per 100 consultations in the first and second year.
Elouafkaoui et al^27^ UK	Cluster Randomized Trial	795 antibiotic prescribing NHS general dental practices in Scotland	*Feedback dashboard* A graphical individualized audit and feedback dashboard for dentists	Dashboard displayed line graph plotting the individual dentist’s monthly antibiotic prescribing rate. Data were derived from 2 routinely collected electronic healthcare datasets held centrally by the Information Services Division of NHS National Services Scotland.	Primary outcome: the total number of antibiotic items dispensed per 100 NHS treatment claims over 12 mo. Secondary outcomes: (1) the defined daily dose (DDD) prescribing rates over 12 mo, (2) the total number of amoxicillin 3g dispensed per 100 NHS treatment claims over 12 mo, and (3) the total number of broad-spectrum antibiotics dispensed per 100 NHS treatment claims over 12 mo.

Dashboards were used as standalone interventions (A&F tools) in 8 studies,^17–20^^,^^22^^,^^24^^,^^26^^,^^27^ while 3 studies evaluated dashboards as a core component of a multifaceted intervention.^21^^,^^23^^,^^25^ In terms of features, all clinical dashboards employed a color-coded system and graphically displayed clinical/patient information (eg, bar chart, line chart, traffic light). Most included studies (n = 10) assessed the effectiveness of dashboards integrated into electronic health/medical record systems.^17–25^^,^^27^ In 2 studies, the dashboards had clinical reminder and alert functions^24^^,^^25^ and 4 used peer comparison by displaying feedback on clinicians’ performance.^17^^,^^18^^,^^22^^,^^27^ The dashboard interventions were predominantly compared with usual care or no intervention in most studies (n = 10),^17–24^^,^^26^ while one study^23^ compared the dashboard with a similar intervention without the dashboard component.

#### Outcomes

Six studies evaluated the changes in medication prescription as the primary outcome,^17^^,^^18^^,^^22^^,^^23^^,^^26^^,^^27^ with one study recording it as the secondary outcome.^21^ Two studies^19^^,^^24^ assessed medication adherence as the primary outcome, while 2 trials focused on test ordering.^20^^,^^21^ One study^25^ had coprimary outcomes on both medication prescription and test ordering.

### Risk of bias

**Figure 2.**
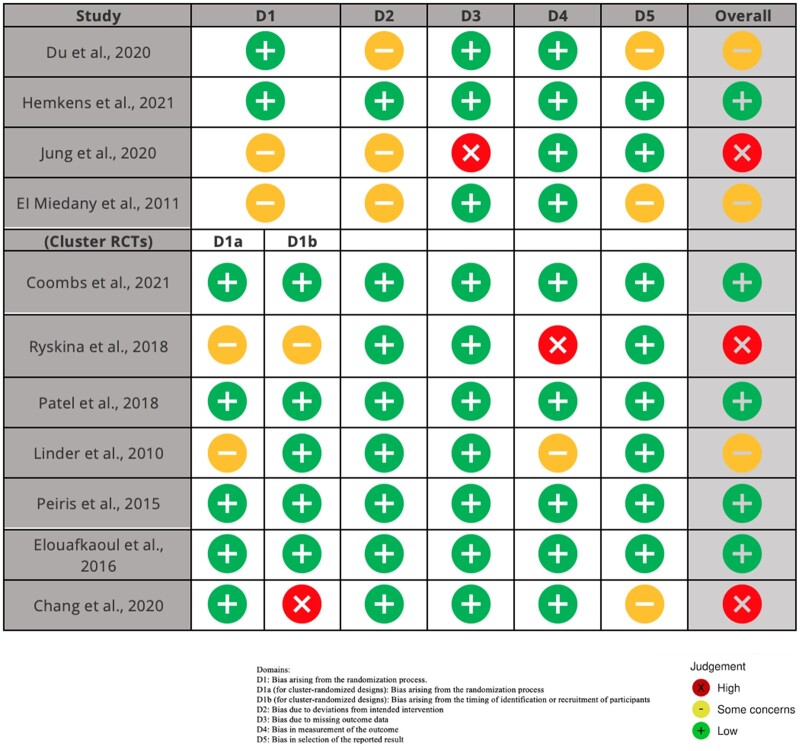
The risk of bias for individual trials. D1: Bias arising from the randomization process. D1a (for cluster-randomized designs): Bias arising from the randomization process. D1b (for cluster-randomized designs): Bias arising from the timing of identification or recruitment of participants. D2: Bias due to deviations from intended intervention. D3: Bias due to missing outcome data. D4: Bias in measurement of the outcome. D5: Bias in selection of the reported result. (X) high risk of bias; (−) some concerns; (+) low risk of bias.

Overall, 4 trials had the risk of bias judged as “some concerns,”^17^^,^^19^^,^^23^ 5 trials had a “low” risk of bias,^18^^,^^21^^,^^25–27^ and 2 trials were considered at “high” risk of bias.^20^^,^^22^^,^^24^ One issue leading to a judgment of an overall “high” risk of bias was a lack of reporting on missing outcome data, and no analysis methods for correcting this bias. In cluster randomized trials, the main issue was the lack of concealment of the cluster allocation, which is likely to lead to selection bias. The risk of bias for individual trials is summarized in Figure 2.

### Quality of the evidence (GRADE)

The quality of evidence is assessed against risk of bias, indirectness, and imprecision. Half of the trials^17^^,^^20^^,^^23^^,^^25^^,^^27^ had a moderate quality of evidence, the common reason for downgrading was due to some concerns within the risk of bias. Three trials^18^^,^^21^^,^^26^ had a high quality of evidence, 1 trial^19^ had a low quality of evidence, while 2 trials^22^^,^^24^ had a very low quality of evidence. The main issue leading to the judgment of low quality was a high risk of bias and uncertainty about imprecision of the estimates due to a lack of effect size reporting.

### Effects of interventions

Table 2 provides the summary of findings for all included studies, including effect sizes (if reported) for both primary and secondary outcomes, the intervention time frame, and the number of participants in each trial. The effects of interventions were classified for dashboards as standalone interventions and dashboards as a core component of a multifaceted intervention, respectively.

**Table 2:** Summary of findings

Study	Time frame		Outcomes		
		Trial groups Intervention (I) Control (C)	Medication prescription/adherence	Test ordering	Quality of the evidence (GRADE)
Computerized feedback dashboard compared with usual care/no intervention (*n* = 8)					
Linder et al^17^ USA	9-mo intervention period	I: 14 primary care practices (258 clinicians, 8406 visits for acute respiratory infection) C: 13 primary care practices (315 clinicians, 10 082 visits for acute respiratory infection)	Antibiotic prescribing rate for all acute respiratory infection visits: OR = 0.97; 95% CI = 0.70–1.40 Antibiotic prescribing rate for antibiotic-appropriate acute respiratory infection visits: I : 63% vs C: 68%; OR = 0.78; 95% CI = 0.53–1.15 Antibiotic prescribing rate for non–antibiotic-appropriate acute respiratory infection visits: I: 32% vs C: 43%; OR = 0.63; 95% CI = 0.45, 0.86	Not reported	Moderate
Patel et al^18^ USA	2-mo intervention period	I (dashboard): 32 physicians and 1743 patients with atherosclerotic cardiovascular disease (ASCVD) I (dashboard with peer comparison): 32 physicians and 1465 patients with ASCVD C: 32 physicians and 1566 patients with ASCVD	Statin prescribing rate for atherosclerotic cardiovascular disease: Dashboard only vs usual care: adjusted difference in percentage points, 4.1; 95% CI = −0.8 to 13.1 Dashboard with peer comparison vs usual care: adjusted difference in percentage points, 5.8; 95% CI, 0.9–13.5	Not reported	High
EI Miedany et al^19^ Europe	6-mo intervention period; (outcome measured over 12 mo)	I: 55 patients diagnosed with early inflammatory arthritis and receiving disease-modifying antirheumatic drug therapy C: 56 patients diagnosed with early inflammatory arthritis and receiving disease-modifying antirheumatic drugs therapy	Medication adherence for disease-modifying antirheumatic drug therapy: I: 87% of patients vs. C: 43% of patients, *P* < .01 (no point estimates and 95% CI reported)	Not reported	Low
Ryskina et al^20^ USA	6-mo intervention period	I: 39 medicine interns and residents C: 34 medicine interns and residents 41 in crossover group: 19 control first then intervention; 22 intervention first then control (114 participants in total)		Count of routine laboratory test orders: −0.14; 95% CI −0.56 to 0.27	Moderate
Chang et al^22^ China	6-mo intervention period	Group 1 (received intervention first then control): 82 primary care physicians Group 2 (received control first then intervention): 81 primary care physicians	10-d antibiotic prescription rate: coef. −0.04, 95% CI −0.07 to −0.01	Not reported	Very low
Jung et al^24^ South Korea	24-wk intervention period	I: 57 kidney transplant recipients C: 57 kidney transplant recipients	Medical adherence among kidney transplant recipients: no significant between-group difference (no point estimates and 95% CI reported)	Not reported	Very low
Hemkens et al^26^ Switzerland	2-y follow-up	I: 1450 primary care physicians C: 1450 primary care physicians	Defined daily doses (DDD) of antibiotic items to any patient per 100 consultations: First year: 0.81%; 95% CI, −2.56% to 4.30% Second year: −1.73%; 95% CI, −5.07% to 1.72%	Not reported	High
Elouafkaoui et al^27^ UK	12-mo intervention period	I: 632 general dental practices (1999 dentists) C: 163 general dental practices (567 dentists)	Number of antibiotic items dispensed per 100 NHS treatment claims: −5.7%; 95% CI −10.2% to −1.1% Defined daily dose (DDD) prescribing rates per 100 NHS treatment claims: −6.6%; 95% CI −12.5% to −0.7% Number of amoxicillin 3g dispensed per 100 NHS treatment claims: −26.0%; 95% CI −64.9% to 13.0% Number of broad-spectrum antibiotics dispensed per 100 NHS treatment claims: −33.3%; 95% CI −80.0% to 20.0%	Not reported	Moderate
Multifaceted interventions incorporating a dashboard component compared with usual care/no intervention (*n *= 2)					
Coombs et al^21^ Australia	4-mo intervention period	I: 4 emergency departments (1392 episodes of care for low back pain) C: 4 emergency departments (3233 episodes of care for low back pain)	Opioid administration rate: OR 0.57; 95% CI 0.38–0.85 Strong opioids administration rate: OR 0.69, 95% CI 0.46–1.04 Nonopioid pain medicines administration rate: OR 1.52, 95% CI 0.98–2.3	Lumbar imaging referral rate: OR 0.77; 95% CI 0.47–1.26	High
Peiris et al^25^ Australia	Median follow-up for intervention and control arms was 17.3 and 17.7 mo	I: 30 general practice services (19 385 patients at high CVD risk) C: 30 general practice services (19 340 patients at high CVD risk)	Appropriate medication prescription rate: RR 1.11; 95% CI, 0.97–1.27 antiplatelet medications: RR 4.80; 95% CI 2.47–9.29 lipid-lowering medications: RR 3.22; 95% CI 1.77–5.88 blood pressure-lowering medications: RR 1.89; 95% CI, 1.08–3.28	Appropriate CVD risk screening rate: RR 1.25; 95% CI 1.04–1.50	Moderate
Multifaceted interventions incorporating a dashboard component compared with similar system without dashboard component (*n* = 1)					
Du et al^23^ USA	11-mo intervention period	I: 22 primary care clinicians C: 23 primary care clinicians	Antibiotic prescription rates for: Upper respiratory infection: ITR 0.60, 95% CI 0.47–0.77 Bronchitis: ITR 0.42, 95% CI 0.32–0.55 Sinusitis: ITR 1.05, 95% CI 0.91–1.21 Pharyngitis: ITR 0.91, 95% CI 0.76–1.09	Not reported	Moderate

#### Clinical dashboards as standalone interventions

Four trials investigated changes in antibiotic prescribing,^17^^,^^22^^,^^26^^,^^27^ with 2 trials having a moderate quality of evidence,^17^^,^^27^ one^26^ having a high quality of evidence and another^22^ having a very low quality of evidence. One trial including 2900 primary care physicians^26^ found a feedback dashboard did not lower nationwide antibiotic prescribing in primary care over 2 years (between-group difference −1.73%, 95% CI −5.07% to 1.72%). Another trial with 573 clinicians also found no effects on antibiotic prescribing for acute respiratory infection (odds ratio 0.97, 95% CI 0.70–1.40).^16^ One trial including 2566 dentists^27^ reported a significant reduction in antibiotic prescribing across all NHS general dental practices (between-group difference −5.7%, 95% CI −10.2% to −1.1%). Another crossover trial^22^ with 163 physicians revealed that a feedback dashboard reduced antibiotic prescribing in primary care by an average of 4% per 10-day period (coef. −0.04, 95% CI −0.07 to −0.01).

One trial^18^ providing a high quality of evidence investigated the effects of a feedback dashboard on statin prescribing for patients with atherosclerotic cardiovascular disease. The trial included 96 primary care physicians and showed no significant difference between the dashboard only intervention arm and the control arm (adjusted difference in percentage points 4.1%, 95% CI −0.8% to 13.1%). However, a significant increase in statin prescribing was seen in the dashboard with peer comparison group (adjusted difference in percentage points 5.8%, 95% CI 0.9% to 13.5%), when compared to the control group.

Patient adherence to prescribed medication was reported in 2 trials with low quality of evidence.^19^^,^^24^ One trial with 111 patients^19^ reported a higher proportion of patients diagnosed with inflammatory arthritis adhering to their prescribed disease-modifying antirheumatic drug therapy in the dashboard group compared to the control group (87% vs 43%, *P* < .01). In another trial^24^ with 114 South Korean kidney transplant recipients, there was no significant between-group difference in adherence to immunosuppressive medications (no effect size provided).

One trial with moderate evidence quality involving 114 medical interns^20^ found no difference in the count of routine laboratory test orders placed by a physician per patient-day in the dashboard intervention group—the ordering dropped by 0.14 less laboratory tests per patient-day among physicians in the intervention group (95% CI −0.56 to 0.27), when compared to the control group.

#### Clinical dashboards as a core component of a multifaceted intervention

One trial providing a high quality of evidence investigated a feedback dashboard as a component of a multifaceted intervention involving staff training and provision of education materials to support guideline-endorsed care of low back pain in emergency departments.^21^ This trial involved 269 clinicians and 4625 patients and found no effects on lumbar imaging referrals (odds ratio 0.77, 95% CI 0.47–1.26), but revealed a significant reduction in opioid administration (odds ratio 0.57, 95% CI 0.38–0.85).

Peiris and colleagues^25^ assessed a multicomponent cardiovascular disease intervention across 60 general practices with 38 725 patients, the quality of evidence was considered moderate. The intervention consisted of computerized decision support, A&F tools, and staff training, where the dashboard was the major component. There was a higher proportion of patients receiving appropriate screening of cardiovascular disease risk factors in the dashboard group versus the control group (risk ratio 1.25, 95% CI 1.04–1.50). This trial^25^ reported no difference in the proportion of patients at high cardiovascular disease risk receiving recommended medication prescription (risk ratio 1.11, 95% CI 0.97–1.27).

One trial with 45 primary care clinicians^23^ found that education plus a feedback dashboard significantly decreased antibiotic prescription rates for upper respiratory tract infections (interaction term ratio 0.60, 95% CI 0.47–0.77) and bronchitis (interaction term ratio 0.42, 95% CI 0.32–0.55), but not for sinusitis (interaction term ratio 1.05, 95% CI 0.91–1.21) and pharyngitis (interaction term ratio 0.91, 95% CI 0.76–1.09), compared to a control group that received education only (moderate quality of evidence).

## DISCUSSION

### Summary of main results

This systematic review assessed the effects of clinical dashboards used as A&F or CDSS on medication prescription, adherence, and test ordering. In standalone interventions, there was conflicting evidence on the effects of dashboards on prescription of antibiotics and statins: 3 trials found no effects on antibiotic or statin prescribing, while 2 trials detected a significant reduction in antibiotic prescribing. Dashboards improved medication adherence in patients with inflammatory arthritis but did not increase the adherence to immunosuppressive medicines in kidney transplant recipients. In multicomponent interventions, dashboards reduced opioid use for low back pain and antibiotic prescribing for upper respiratory tract infections. For test ordering, dashboards increased the proportion of patients receiving appropriate cardiovascular risk screening but had no effect on the rate of imaging referrals for low back pain.

### Comparison with existing studies

A previous narrative systematic review investigating the effects of dashboards was conducted by Dowding and colleagues in 2015.^5^ Eleven studies were identified with empirical evaluations of dashboards; however, the majority of the studies were nonrandomized pre-post designs with only 1 randomized controlled trial included. Whilst our review focused on investigating dashboards on medication use and test orders through a comprehensive review of randomized controlled studies, we included 11 relevant trials. Another systematic review on the effectiveness of information display interventions on patient care outcomes was published in 2019.^12^ Of 22 eligible studies included, only 5 were randomized controlled studies. The findings suggested that there was limited evidence that dashboards significantly improve patient outcomes. However, this systematic review was conducted solely in critical care settings and included various information display interventions, such as physiologic and laboratory monitoring, expert systems, and multipatient dashboards. Notably, there was a recent review on patient safety dashboards published in late 2021.^28^ It analyzed 33 time-series studies and case studies and concluded limited evidence for dashboards directly or indirectly impacting patient safety.

Clinical dashboards are not only used as standard standalone interventions but are also frequently used as part of multifaceted interventions for improving healthcare performance and processes. Our review found the effects vary greatly in terms of medication prescription, medication adherence, and test ordering. This high level of heterogeneity aligns with findings from other systematics reviews evaluating the impacts of electronic A&F^29^ and CDSS^30^^,^^31^ without the dashboard component. The heterogeneity of our findings might result from dashboards being adopted using different formats, using different technologies, in different health settings, for different end-users. In the included studies of our review, intervention effects were assessed either solely on the dashboard itself or by incorporating dashboards into multicomponent interventions. Therefore, it is difficult to arrive at a definitive conclusion why some dashboard interventions contribute to improvements in healthcare performance while others do not.

### Explanations and implications for future research

One issue leading to a lack of significant intervention effects might be related to data quality. This is evidenced by one trial^26^ that failed to reduce the antibiotic prescription rate, where the authors discussed that incompleteness of the routine health data may have hindered comprehensive feedback on prescribing rates to clinicians. The issues of accuracy, completeness, interoperability, and reliability that are associated with routine health data have been widely acknowledged and yet no perfect solutions have been brought forward. Therefore, provided that the digital dashboard, by its nature, is a data visualization and analytics tool, it would inevitably suffer from the deficiencies of healthcare data. This may result in the inability of digital health dashboards to accurately display a full picture of patient information or healthcare use, which might impede effective feedback and decision-making.

Another possible reason for the nonsignificant effects might be a lack of dashboard use by clinicians or patients.^32^ For effective implementation of dashboards into routine care there is a need for a thorough inspection of the organizational environment preimplementation. When implementing health informatics tools such as clinical dashboards, healthcare organizations should take multilevel factors into account, such as people, process, technology, and their interactions,^33^ to identify factors that might impede the health interventions from achieving their full potential.^34^ That is, to implement a health technology, not only should evaluation efforts focus on the intervention itself, but they should also consider the underlying infrastructure that supports the devices, and the human factors such as clinicians’ readiness and digital literacy, as well as the healthcare organizational environment and resources for properly implementing the technology.

Behavior change theories are encouraged to be used when designing interventions aiming to change clinicians’ practice, including clinical dashboards.^11^^,^^35^ Recently, Dowding and colleagues^36^ proposed a theory to guide the design of clinical dashboards, including 3 domains: the cues of the intervention message; the nature of the task or behavior to be performed; and situational/personality variables. Cues of the intervention message focus on providing specific tasks and performance goals as opposed to more generalized feedback.^36^ One trial in our review^27^ designed an A&F dashboard for dentists using a similar behavior change technique involving “instructions on how to perform the behavior” and “provision of information about health consequences of performing the behavior.” The authors found that this dashboard led to a significant reduction in antibiotic prescribing.^27^ The nature of the task to be performed concerns cognitive resources—the more cognitively demanding a task is, the less effective the intervention would be.^36^ Another trial in our review^17^ developed a quality dashboard for acute respiratory infections, which included data unrelated to study outcomes (eg, distribution of patient visits, billing information) displayed with 10 other reports unrelated to the trial’s activities. The high cognitive demand tasks associated with this dashboard could explain the lack of effects of the intervention on antibiotic prescribing for acute respiratory infections.^17^ Situational/personality factors relate to baseline performance.^36^ In one of our included trials,^24^ the authors claimed that the nonsignificant improvement in medication adherence was in part due to already high baseline adherence. Another trial^26^ also explained that the feedback dashboard was not associated with reduced prescribing rates possibly because Switzerland has the lowest antibiotic prescription rates in Europe. The same factor was observed in the SHaPED trial,^21^ which had low preintervention lumbar imaging rates. Thus, given the already high-level baseline performance, it would be more challenging for dashboard intervention to make a difference. None of the other trials included in this review explicitly stated that they have used theory to design dashboard components.

With the capability of integrating and sharing real-time health data, digital health dashboards are deemed as an important building block for developing a learning health system.^37^^,^^38^ A learning health system learns from routine health data and feeds the evidence back into practice to create cycles of continuous improvement.^37^ Within this process, the dashboard has therefore moved from a one-way linear data output model (health data input—dashboard—analyzed data presentation) to a cyclical data output model (data input from electronic health record—dashboard and other health technologies—integrated data presentation—update data into electronic health record). By harnessing the power of capturing real-time health data, and integrating data from various sources, digital health dashboards can be a critical enabler in accelerating the uptake of evidence into practice, thereby improving healthcare performance, patient safety, and quality of care. This significance has been increasingly recognized especially during the COVID-19 pandemic.^39^ With the urgent need to timely acquire patient’s demographics, COVID-19 severity, risk factors, and test results, population health dashboards/national dashboards have been developed to monitor pandemics and assist in making clinical decisions and public health policies.^40^ The evidence generated from disease diagnosis and management is then updated in the routine healthcare databases and shown in the dashboards to better inform practice. This learning health system has its unique role in promising us to rapidly adapt to public health emergencies, and the dashboard is no doubt a key enabler.

### Strengths and limitations

The strengths of this study include the adoption of a robust methodology for systematically searching, screening, extracting, and summarizing the existing evidence. Meanwhile, we only included randomized controlled trials which helped to reduce the heterogeneity of included studies. Nevertheless, there was still a high level of heterogeneity regarding the study populations, health conditions, and outcomes measures in included trials, which is the main limitation of this review. Secondly, there is a deviation from the study protocol,^14^ where we initially planned to consider a wide range of study designs since the previous review on this topic included only 1 randomized trial. However, in our searches we were able to find 11 eligible randomized trials investigating the effects of dashboards, which allowed us to focus on the highest level of evidence. We also narrowed down the outcome measures into medication use and test orders since these are relevant outcomes related to quality and safety of healthcare and more likely to be influenced by dashboard interventions. We believe that we did not miss any relevant trials considering our sensitive search strategy and we manually screened potentially eligible studies cited in the included studies. Finally, as there were 2 dashboard types assessed in the review, when it comes to multifaceted interventions, assigning specific effects to the dashboard component inevitably became equivocal.

## CONCLUSION

There is limited evidence indicating the positive impact of introducing clinical dashboards into routine practice on medication use and test ordering. Dashboards seem to have become an integral component of healthcare organizations with a prior assumption that they are useful, but the evidence from our review contradicts this assumption to some extent. When designing and implementing dashboards in healthcare, important aspects, such as design theories, data quality, healthcare processes, human factors and available resources, warrant further attention as they might influence the effects of dashboards on healthcare performance and quality of care.

## AUTHOR CONTRIBUTIONS

CX, GCM, and CGM conceived the study. QC, LH, and CH supported the data extraction. All authors contributed to the planning of the study and the manuscript.

## SUPPLEMENTARY MATERIAL

Supplementary material is available at *Journal of the American Medical Informatics Association* online.

## PATIENT AND PUBLIC INVOLVEMENT

There was no involvement from patients or the public in the design, conduct, or outcome of this work.

## CONFLICT OF INTEREST STATEMENT

None declared.

## Supplementary Material

ocac094_Supplementary_Data
