# Supplementary material for: Effectiveness of clinical dashboards as audit and feedback or clinical decision support tools on medication use and test ordering: a systematic review of randomized controlled trials
Source: J Am Med Inform Assoc. 2022 Jun 11;29(10):1773–85. doi: 10.1093/jamia/ocac094 (PMC9471705; doi:10.1093/jamia/ocac094)
Supplement: ocac094_Supplementary_Data [file ocac094_supplementary_data.docx]

**Supplementary File 1. Search Strategy Summary**

**Search Databases:**

MEDLINE via Ovid, EMBASE via Ovid, and CINAHL via EBSCO, CENTRAL via Cochrane Library, INSPEC, ACM Digital Library, and IEEEXplore

1. **MEDLINE via Ovid**
2. Information Science/ or Data Display/ or Data Science/ or Information Technology/
3. Decision Support Systems, Clinical/ or Health Information Systems/ or Integrated Advanced Information Management Systems/ or Hospital Information Systems/
4. exp Electronic Health Records/ or Medical Records Systems, Computerized/ or Health Smart Cards/ or Decision Making, Computer-Assisted/ or User-Computer Interface/
5. Quality Indicators, Health Care/ or Benchmarking/ or Physician's Practice Patterns/
6. (electronic health record* or electronic medical record* or EHR or EMR or clinical information system* or health information technolog*).ti,ab.
7. 1 or 2 or 3 or 4 or 5
8. (dashboard* or data visuali?ation or data analytic* or business intelligence or feedback system or electronic feedback).mp.
9. 6 and 7
10. **EMBASE via Ovid**
11. Information Science/ or Data Display/ or Data Science/ or Information Technology/
12. Decision Support Systems, Clinical/ or Health Information Systems/ or Integrated Advanced Information Management Systems/ or Hospital Information Systems/
13. exp Electronic Health Records/ or Medical Records Systems, Computerized/ or Health Smart Cards/ or Decision Making, Computer-Assisted/ or User-Computer Interface/
14. Quality Indicators, Health Care/ or Benchmarking/ or Physician's Practice Patterns/
15. (electronic health record* or electronic medical record* or EHR or EMR or clinical information system* or health information technolog*).ti,ab.
16. 1 or 2 or 3 or 4 or 5
17. dashboard*.mp. [mp=title, abstract, heading word, drug trade name, original title, device manufacturer, drug manufacturer, device trade name, keyword, floating subheading word, candidate term word]
18. 6 and 7
19. **CINAHL via EBSCO**
20. information science OR information technology OR data display OR data science
21. decision support system OR (decision making or decision-making or decision making process or decision-making process ) OR health information systems OR information management system OR hospital information systems
22. TI, AB (electronic health record* OR (medical records or health records or medical reports or medical information) OR user computer interface OR electronic medical record* OR EHR OR EMR OR clinical information system* OR health information technolog*)
23. quality indicators in healthcare OR benchmarking in healthcare OR Physician's Practice Patterns
24. OR (1-4)
25. dashboard* OR data visuali?ation OR data analytic* OR business intelligence OR feedback system OR electronic feedback
26. 5 AND 6
27. **CENTRAL via Cochrane Library**
    1. MeSH descriptor: [Information Science] explode all trees
    2. MeSH descriptor: [Decision Support Systems, Clinical] explode all trees
    3. MeSH descriptor: [Electronic Health Records] explode all trees
    4. MeSH descriptor: [Quality Indicators, Health Care] explode all trees
    5. MeSH descriptor: [Practice Patterns, Physicians'] explode all trees
    6. 1 or 2 or 3 or 4 or 5
    7. (dashboard* or data visuali?ation or data analytic* or business intelligence or feedback system or electronic feedback):ti,ab,kw
    8. 6 and 7
28. **INSPEC**

(( ((dashboard*) WN ALL) AND (1896-2020 WN YR)) AND ( (((((({decision support systems} WN CV) OR ({management information systems} WN CV) OR ({decision making} WN CV) OR ({information systems} WN CV) OR ({medical information systems} WN CV)))) AND (1896-2020 WN YR)) OR ((($data $science OR $data $display) WN ALL) AND (1896-2020 WN YR)) OR ((((({information science} WN CV) OR ({information analysis} WN CV) OR ({information services} WN CV) OR ({information management} WN CV) OR ({information retrieval} WN CV) OR ({information retrieval systems} WN CV) OR ({information systems} WN CV) OR ({information technology} WN CV) OR ({information use} WN CV)))) AND (1896-2020 WN YR)) OR (((((({benchmark testing} WN CV)))) OR ((quality indicator*) WN ALL) OR (({Physician's Practice Patterns}) WN ALL) OR ((benchmark$) WN ALL)) AND (1896-2020 WN YR)) OR (((((({electronic health records} WN CV) OR ({medical information systems} WN CV) OR (clinical information system* WN KY) OR (health information technolog* WN KY) OR ({EHR} WN KY) OR ({EMR} WN KY))))) AND (1896-2020 WN YR)))))

1. **ACM Digital Library**

[[All: [[information science]] OR [All: [data display]] OR [All: [data science]] OR [All: [information technology]]] OR [All: [[clinical decision support system*]] OR [All: [health information system*]] OR [All: [integrated advanced information management system*]] OR [All: [hospital information system*]]] OR [All: [[electronic health record*]] OR [All: [medical records system*]] OR [All: [health smart card*]] OR [All: [decision making]] OR [All: [user-computer interface]]] OR [All: [[quality indicators]] OR [All: [benchmark*]] OR [All: [physician's practice patterns]]] OR [All: [[electronic health record*]] OR [All: [electronic medical record*]] OR [All: [ehr]] OR [All: [emr]] OR [All: [clinical information system*]] OR [All: [health information technolog*]]] OR [All: [[data visuali?ation]] OR [All: [data analytic*]] OR [All: [business intelligence]] OR [All: [feedback system]] OR [All: [electronic feedback]]]] AND [Abstract: dashboard*]

1. **IEEE Xplore**

((((("Full Text & Metadata":Information Science OR Data Display OR Data Science OR Information Technology) OR "Full Text & Metadata":Clinical Decision Support Systems OR Health Information Systems OR Integrated Advanced Information Management Systems OR Hospital Information Systems OR Clinical Information Systems OR Health Information Technolog*) OR "Full Text & Metadata":Electronic Health Records OR Electronic Medical Records OR Medical Records Systems OR Health Smart Cards OR Decision Making OR User-Computer Interface OR EHR OR EMR ) OR "Full Text & Metadata":Quality Indicators OR Benchmarking OR Physician's Practice Patterns) AND "All Metadata":dashboard*)
